# Petri net-based prediction of therapeutic targets that recover abnormally phosphorylated proteins in muscle atrophy

**DOI:** 10.1186/s12918-018-0555-0

**Published:** 2018-03-05

**Authors:** Jinmyung Jung, Mijin Kwon, Sunghwa Bae, Soorin Yim, Doheon Lee

**Affiliations:** 1 Bio-Synergy Research Center, 291 Daehak-ro, Yuseong-gu, 305-701 Daejeon, Republic of Korea; 2 Department of Applied Statistics, College of Economics and Business, The University of Suwon, Hwaseong-si, Gyeonggi-do, 18323 Republic of Korea; 3 Department of Bio and Brain Engineering, KAIST, 291 Daehak-ro, Yuseong-gu, Daejeon, 305-701 Republic of Korea

**Keywords:** Muscle atrophy, Therapeutic target, Petri net, Phosphorylation

## Abstract

**Background:**

Muscle atrophy, an involuntary loss of muscle mass, is involved in various diseases and sometimes leads to mortality. However, therapeutics for muscle atrophy thus far have had limited effects. Here, we present a new approach for therapeutic target prediction using Petri net simulation of the status of phosphorylation, with a reasonable assumption that the recovery of abnormally phosphorylated proteins can be a treatment for muscle atrophy.

**Results:**

The Petri net model was employed to simulate phosphorylation status in three states, i.e. reference, atrophic and each gene-inhibited state based on the myocyte-specific phosphorylation network. Here, we newly devised a phosphorylation specific Petri net that involves two types of transitions (phosphorylation or de-phosphorylation) and two types of places (activation with or without phosphorylation). Before predicting therapeutic targets, the simulation results in reference and atrophic states were validated by Western blotting experiments detecting five marker proteins, i.e. RELA, SMAD2, SMAD3, FOXO1 and FOXO3. Finally, we determined 37 potential therapeutic targets whose inhibition recovers the phosphorylation status from an atrophic state as indicated by the five validated marker proteins. In the evaluation, we confirmed that the 37 potential targets were enriched for muscle atrophy-related terms such as actin and muscle contraction processes, and they were also significantly overlapping with the genes associated with muscle atrophy reported in the Comparative Toxicogenomics Database (*p*-value < 0.05). Furthermore, we noticed that they included several proteins that could not be characterized by the shortest path analysis. The three potential targets, i.e. BMPR1B, ROCK, and LEPR, were manually validated with the literature.

**Conclusions:**

In this study, we suggest a new approach to predict potential therapeutic targets of muscle atrophy with an analysis of phosphorylation status simulated by Petri net. We generated a list of the potential therapeutic targets whose inhibition recovers abnormally phosphorylated proteins in an atrophic state. They were evaluated by various approaches, such as Western blotting, GO terms, literature, known muscle atrophy-related genes and shortest path analysis. We expect the new proposed strategy to provide an understanding of phosphorylation status in muscle atrophy and to provide assistance towards identifying new therapies.

**Electronic supplementary material:**

The online version of this article (10.1186/s12918-018-0555-0) contains supplementary material, which is available to authorized users.

## Background

Muscle atrophy is defined as the involuntary loss of muscle mass, resulting in low quality of life, morbidity and mortality [1]. It is associated with various diseases, such as heart failure [2], chronic kidney disease (CKD) [3] and cancer-cachexia [4], that occur in 80% of patients with advanced cancer [5]. In recent years, many researchers have been devoted to discovering drugs for muscle atrophy. For example, Enobosarm, launched by the biotech firm GTx in 2011, is a molecule that binds to the testosterone receptor in the muscle in order to stimulate muscle build-up [5]. Bimagrumab is an anti-myostatin antibody developed by Novartis that prevents myostatin (one of the main factors inducing muscle atrophy) from activating the ACTRIIB receptor [1]. Unfortunately, these drugs have shown limited effects on muscle atrophy [5, 6], and therefore new therapeutic strategies are required for treating muscle atrophy. Here, we present a new systematic approach to predict therapeutic targets for muscle atrophy.

For therapeutic target prediction, the effects of gene inhibition (knock-down) are necessarily analyzed with a general assumption that most drugs inhibit their targets. shRNA knockdown, one of the in-vitro experiments, is a popular and typical tool for the purpose. However, it is time-consuming and labor-intensive to perform the experiments in genome-scale, especially with a specific disease condition (e.g. atrophic state in this case). Thus, in this study, we employed the Petri net, an in-silico method, which enables dynamic simulations on a large-scale network with incomplete kinetic parameters [7]. The Petri net facilitates genome scale analysis with low cost and short time. Due to this beneficial characteristic, Petri net has been frequently used to model and simulate perturbations of biological signaling networks in previous studies. For example, the Lee D group applied efficient dynamic simulation of EGF-induced signal transduction pathways with colored Petri net [8]. In Derek Ruths’ work, the signaling Petri net-based simulator was employed for the MAPK and AKT signaling network in breast cancer cell lines for computing the response to TSC2 and mTOR inhibitions [9]. In addition, the Jin group proposed an enhanced Petri net model to predict the synergistic effects of drug combinations, which was simulated for signaling pathways and protein-protein interactions related to EGFR and BCL2 [10].

**Fig. 1:**
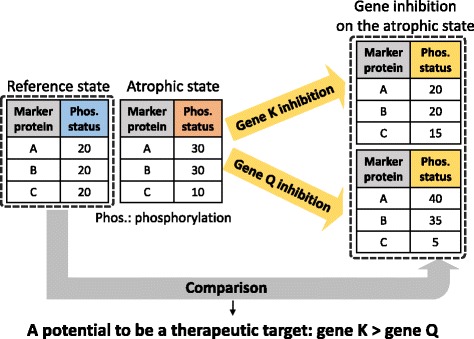
Strategy for identifying potential therapeutic targets for muscle atrophy. We have a list of marker proteins showing different phosphorylation status in atrophic compared to reference states. Gene K inhibition on the atrophic state recovers their phosphorylation status to that of reference state, whereas gene Q inhibition deepens the phosphorylation status to that of atrophic state. Thus, in this example, gene K has more potential to be a therapeutic target for muscle atrophy

In this study, we focused on phosphorylation status in an atrophic state. Phosphorylation plays one of the key regulatory roles in signal transduction by sequentially activating/deactivating various proteins and enabling cells to respond to external stimuli [11]. Due to their important roles, abnormally phosphorylated proteins are involved in many diseases [12, 13]. In muscle atrophy, as well, some proteins are abnormally phosphorylated, such as FOXO and SMAD [14, 15]. Here, we assumed that muscle atrophy could be treated if abnormally phosphorylated proteins in the atrophic state are recovered. Therefore, in this study, a potential therapeutic target for muscle atrophy is defined as a gene whose inhibition is capable of recovering phosphorylation status from the atrophic state, which is depicted in Fig. 1.

We predicted therapeutic targets for muscle atrophy by analyzing phosphorylation status among reference, atrophic and each gene-inhibited state, which were computed by Petri net simulations on large-scale phosphorylation network. Here, we developed a phosphorylation-specific Petri net model whose token represents the amount of phosphate and that involves two types of transitions (phosphorylation or de-phosphorylation) and two types of places (activation with or without phosphorylation). The difference between the phosphorylation status of reference and atrophic states were validated by Western blotting experiments detecting five marker proteins, i.e. RELA, SMAD2, SMAD3, FOXO1 and FOXO3. Among 331 kinases and phosphatases, all of which link to the five validated marker proteins in the integrated network, we generated 37 potential therapeutic targets whose inhibition recovers the five abnormally phosphorylated proteins in atrophic states. The predicted therapeutic targets were validated by the genes associated to muscle atrophy reported in the Comparative Toxicogenomics Database (CTD) [16], and they were compared with that of the shortest path analysis. Furthermore, they were evaluated by the enriched gene ontology terms and KEGG pathways, and three of them (i.e. BMPR1B, ROCK, and LEPR) were verified by literature evidence.

## Methods

### Methods overview

**Fig. 2:**
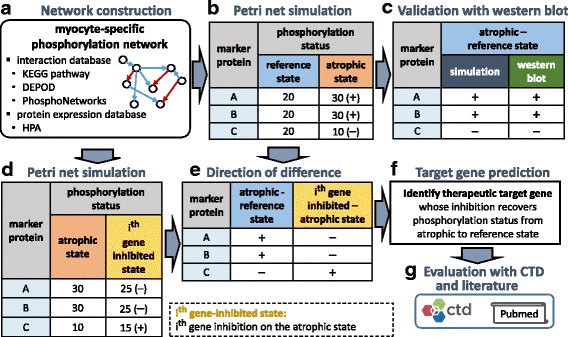
Methods overview

A myocyte-specific phosphorylation network consisting of 1985 nodes (protein) and 7283 edges (phosphorylation and de-phosphorylation) were constructed from four public databases, i.e., KEGG pathway [17], DEPOD [18], PhosphoNetworks [19] and Human Protein Atlas [20] (Fig. 2a). Firstly, on the myocyte-specific phosphorylation network, the phosphorylation status of reference and atrophic states were computed by employing a phosphorylation-specific Petri net model devised in this study (Fig. 2b). Then, the saturated phosphorylation status of the pre-determined marker proteins, which are known to be abnormally phosphorylated in the atrophic state, were validated by Western blotting experiments (Fig. 2c). For therapeutic target prediction, we additionally performed 331 Petri net simulations on the atrophic state with each gene inhibition (Fig. 2d, i^th^ gene-inhibited state). Then, we selected proteins whose inhibition recovers the phosphorylation status from atrophic to reference state in terms of the five validated proteins in (Fig. 2c, e, and f). Finally, the predicted therapeutic targets were evaluated using the genes associated to muscle atrophy reported in CTD and by literature evidence (Fig. 2g).

### Phosphorylation network construction

We constructed an integrated phosphorylation network, whose nodes represent proteins and whose edges represent either phosphorylation or de-phosphorylation. To this end, we retrieved phosphorylation and de-phosphorylation interactions from three databases, the Kyoto Encyclopedia of Genes and Genomes (KEGG) pathway [17], the Dephosphorylation Database (DEPOD) [18] and PhosphoNetworks [19]. KEGG pathway is a collection of manually drawn pathways representing various molecular interaction and reactions, where 3529 phosphorylation and 1140 de-phosphorylation interactions were extracted. DEPOD is a manually curated de-phosphorylation database collecting interactions between human phosphatases and their substrates, where 888 de-phosphorylation interactions were extracted. PhosphoNetworks contains kinase-substrate relationships retrieved from recent high-throughput protein microarrays as well as manually curated literature, where 4337 phosphorylation interactions were extracted.

**Fig. 3:**
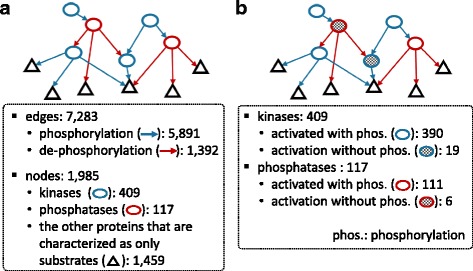
Network configuration. **a** Statistics of the edges and nodes in the network (**b**) Statistics of the kinases and phosphatases, which are a part of the nodes, according to an activation condition

The kinases, phosphatases and their substrate proteins in the extracted interactions were mapped to Entrez ID. We deleted 215 duplicated interactions and removed 10 conflicted interactions with literature evidence, which resulted in 9669 interactions (7737 phosphorylation and 1932 de-phosphorylation interactions) among 2326 proteins. In order to consider muscle cell (myocyte)-specific conditions on the constructed network, we removed interactions that included non-detected proteins in myocytes reported in The Human Protein Atlas [20]. This finally resulted in 7283 interactions (5891 phosphorylation and 1392 de-phosphorylation interactions) among 1985 proteins, which consist of 409 kinases, 117 phosphatases and the other 1459 proteins that are not characterized as kinases and phosphatases but only their substrates. The statistics are summarized in Fig. 3a.

A phosphorylation or de-phosphorylation event occurs when the enzyme, such as kinase or phosphatase, is activated. We needed to determine the activation condition of the 409 kinases and 117 phosphatases in terms of their phosphorylation status because we utilize phosphorylation status, instead of activity status, in the Petri net model. We basically assume that most kinases and phosphatases are activated with phosphorylation. This is based on several reports in the literature showing that phosphorylation leads to an activation of the substrate [21, 22] and an external stimuli leads to the consecutive activation of several down-stream kinases with phosphorylation (phosphorylation cascade) [23]. However, we do not believe that all kinases and phosphatases are activated with phosphorylation. Among the 409 kinases and 117 phosphatases, we characterized 19 kinases and 6 phosphatases that are activated without phosphorylation (i.e. inhibited with phosphorylation) based on the information in KEGG pathways. Here, we determined that an enzyme K is activated without phosphorylation if both *activation* and *de-phosphorylation* or both *inhibition* and *phosphorylation* are concurrently assigned for a certain enzyme Q to K in KEGG pathways. The other 390 kinases and 111 phosphatases were regarded to be activated with phosphorylation based on the assumption (Fig. 3b).

### Petri net configuration

In the Petri net model used in this study, the proteins are represented by places (1985 places), and phosphorylation and de-phosphorylation interactions are represented by transitions (7283 transitions), and the amount of phosphates is represented by the number of tokens, an integer. The amount of phosphates in a specific protein (the phosphorylation status) is represented by the number of tokens in the corresponding place, whose value is an integer that is equal to or greater than zero. For the purpose of the study, we developed a phosphorylation-specific Petri net configuration that involves two types of transitions and two types of places, which is quite different from a typical Petri net model.

**Fig. 4:**
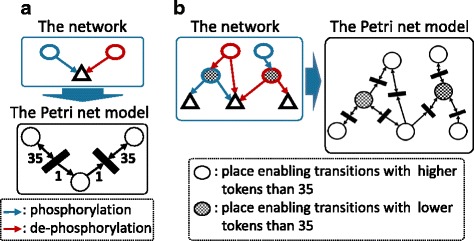
Petri net configuration for the two types of transitions and the two types of places. **a** Two types of transitions in the Petri net model that depict phosphorylation and de-phosphorylation. **b** Two types of places in the Petri net model that apply different enabling conditions

There are two types of transitions, phosphorylation (5891 transitions) and de-phosphorylation (1392 transitions), whose corresponding Petri net transitions were depicted in Fig. 4a. Basically, the number of tokens in an input place is not changed by any firing transition, which reflects that the phosphorylation reaction of an enzyme (kinase or phosphatase) does not change the amount of phosphates of itself. On the other hand, the number of tokens in an output place increases by one when a phosphorylation is fired, and it decreases by one when a de-phosphorylation is fired. It reflects real phosphorylation/de-phosphorylation that attach/remove phosphates to/from a substrate. The number of tokens enabling transitions (enabling threshold) was determined as 35, which is the most robust among eleven candidate thresholds (refer to the enabling threshold of the Petri net model in Methods).

We also presented the two types of places as introduced in the above section. It should be noted that the criteria for the two types of places is an activation condition of the enzymes, such as activation with phosphorylation or without phosphorylation, and not a function of the enzymes, such as kinase or phosphatase that has been already considered in transitions. For an input place that is activated with phosphorylation (309 kinases and 111 phosphatases), its transition is enabled when the number of tokens in the input place is greater than the enabling condition, 35. On the other hand, for an input place that is activated without phosphorylation (19 kinases and 6 phosphatases), its transition is enabled when the number of tokens in the input place is less than 35 (Fig. 4b).

### Petri net simulation

**Fig. 5:**
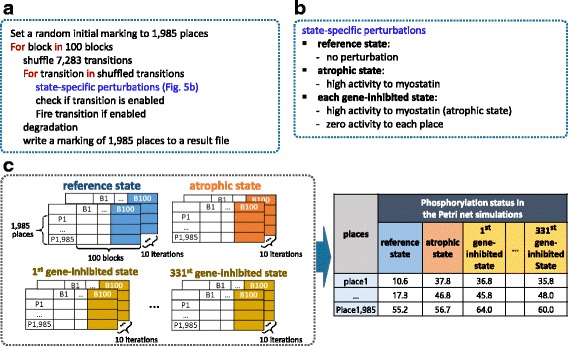
A high-level procedure of Petri net simulations for three states and their results. **a** Common procedures for all of three states except for state-specific perturbations. **b** State-specific perturbations for each of three states. Before checking each transition, we give perturbations to relevant places according to each state. For the reference state, there is no perturbation. For the atrophic state, high activity is assigned to myostatin that induces muscle atrophy. For each gene-inhibited state, zero activity is assigned to each place on the atrophic state. **c** Simulation results. The final markings (the marking of 100th block) of the ten iterations were averaged to get the phosphorylation status for one reference, one atrophic and 331 gene inhibited states

We performed Petri net simulations on the phosphorylation network for three states, i.e. reference, atrophic and each gene-inhibited state. To reduce computation time in the massive simulations on the large-scale network, we partially adopted the Petri net simulation procedure presented in Derek Ruths’ work [9]. In one Petri net simulation, we first set a random value between zero and twice the enabling threshold (i.e. 70) to every place, which is a random initial marking, where a marking is a distribution of tokens over all places. Then, for each 100 blocks, we generate a list of transitions in a random order, i.e., a shuffled transition list. For each transition in the shuffled transition list, we apply state-specific perturbations (Fig. 5b) and check if the transition is enabled, followed by firing the transition if it is enabled (Fig. 5a).

In state-specific perturbations, there is no perturbation for the reference state. On the other hand, for atrophic state, high activity is assigned to myostatin that is a protein inhibiting muscle cell growth and inducing muscle atrophy [24]. This perturbation is consistent with the Western blotting condition that continuously treats the myostatin to normal muscle for its transition to atrophic muscle. Due to the absence of myostatin from the network, we assigned high activity to its direct targets instead of myostatin itself. In each gene-inhibited state, we set zero activity to each place on the atrophic state (Fig. 5b). Here, among 526 phosphorylation-related enzymes (409 kinases and 117 phosphatases), we only performed 331 gene-inhibited state simulations for 256 kinases and 72 phosphatases that are linked to the five validated markers in wet experiments (see Intermediate validation of the Petri net model in Methods). After performing all transitions in the shuffled transition list, we executed a biological degradation process by reducing 10% of the tokens in every place and write the resulting marking to a file.

Due to randomness in assigning the initial marking and shuffling transition list, 10 Petri net simulations were performed for the reference, atrophic and each gene-inhibited state, respectively (10 iterations). We noticed that tokens were saturated at the final block (the 100th block) for most places in all three states, and for each state we computed the phosphorylation status of 1985 proteins by averaging the final markings (the marking of 100th block) of the 10 iterations. As a result, we obtained the phosphorylation status of 1985 proteins in one reference, one atrophic, and 331 gene inhibited states (Fig. 5c). All of the Petri net simulations were performed by Python codes developed in this study.

### Intermediate validation of the petri net model

**Fig. 6:**
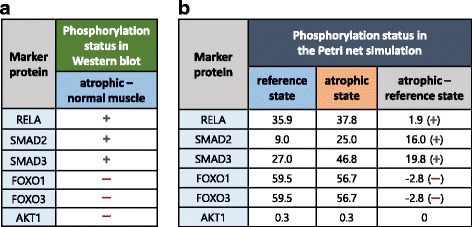
The changes in phosphorylation status for six marker proteins. **a** The changes measured in Western blotting between normal and atrophic muscle. **b** The changes calculated in Petri net simulation between reference and atrophic state. We identified the coincident directions of changes in the five proteins (RELA, SMAD2, SMAD3, FOXO1, and FOXO3)

From three Western blotting experiment papers [15, 25–27], atrophy marker proteins were collected as six proteins (RELA, SMAD2, SMAD3, FOXO1, FOXO3 and AKT1) showing different phosphorylation states between normal and myostatin-induced atrophic muscle. The myostatin-induced atrophic muscle is induced by treatment with myostatin to normal muscle in a certain period, which is consistent with the Petri net configuration for atrophic state that gives high activity to the myostatin place. The Western blotting results show that three proteins are hyper-phosphorylated (RELA, SMAD2, SMAD3) and the others are hypo-phosphorylated (FOXO1, FOXO3, AKT1) in an atrophic muscle compared to normal muscle (Fig. 6a).

For the six marker proteins, the differences in the simulation results between reference and atrophic state were compared to those of the Western blotting experiments in Fig. 6a. Here, we did not focus on the amount of changes, but rather the directions of the changes. As a result, we observed the same directions of changes in the five proteins (RELA, SMAD2, SMAD3, FOXO1, and FOXO3) except for the AKT1 protein (Fig. 6b). An imperfection in the phosphorylation network might be one of the reasons for the inconsistency with AKT1 protein. With these coincident directions in the five proteins, we decided to use the five validated proteins as the marker proteins to predict potential of therapeutic targets. As mentioned in the above section, Petri net simulations in the gene-inhibited state were only performed for 331 genes that link to these five validated markers.

### Enabling threshold of the petri net model

The Petri net simulation results highly depend on the enabling threshold we selected. Thus, we selected the most robust threshold, i.e. 35, among the eleven enabling thresholds (1, 5, 10, 15, 20, 25, 30, 35, 40, 45, and 50). To do this, for each enabling threshold, ten simulations were performed in reference and atrophic states, respectively, with consideration of randomness in assigning initial marking and shuffling transition list. Then, for each simulation, we generated a list of the ranked places by their final marking (the marking of 100th block), which describes the saturated phosphorylation status. Among the ten list of the ranked places in an enabling threshold, we calculated rank correlations in a pair-wise manner with the spearman rank correlation, which results in 45 rank correlations (except for the correlation of itself). When averaging the 45 rank correlations, the enabling threshold of 35 yields the highest rank correlation (0.791), which indicates that the threshold of 35 is the most robust among the eleven thresholds. Thus, we decided to use 35 as the enabling threshold (Additional file 1: Figure S1).

### Therapeutic target prediction

**Fig. 7:**
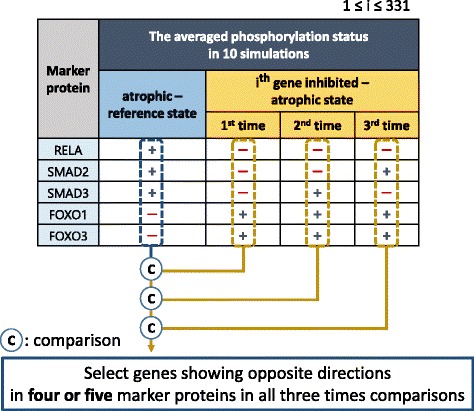
Selection process for potential therapeutic target prediction

As the strategy introduced in Fig. 1, a potential therapeutic target for muscle atrophy is defined as a gene whose inhibition is capable of recovering phosphorylation status from an atrophic state with respect to the five validated marker proteins. To do this, for the five validated marker proteins, we measured the directions of changes from the reference to atrophic state, and we also computed the directions of changes from the atrophic to each of 331 gene-inhibited states (Fig. 7). Then, by comparing the two types of directions, we selected the genes showing opposite directions in four or five marker proteins among the five. To get more robust results, we executed each gene-inhibited state three times, i.e., totaling 331*10*3 simulations, and we finally selected the genes showing opposite directions in four or five marker proteins in all three comparisons (Fig. 7).

## Results

### Characterized therapeutic targets

**Fig. 8:**
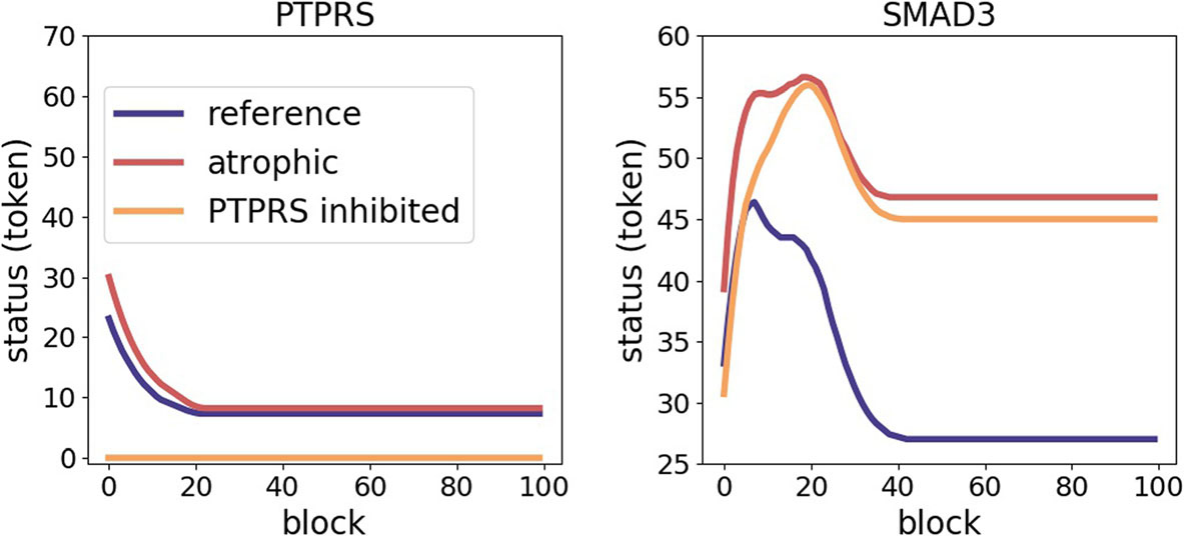
Plot of the simulation results in the reference, atrophic, and PTPRS-inhibited states. We only depicted two proteins, i.e., SMAD3 (one of the five marker proteins) and PTPRS (inhibited protein), to avoid a crowded figure. The status of SMAD3 becomes lower in the PTPRS-inhibited than the atrophic state that indicates the recovery from the atrophic to reference state, which is a condition for a therapeutic target in this study

With the process introduced in the Methods, among 331 candidate enzymes, we finally characterized 37 therapeutic targets (30 kinases and 7 phosphatases) whose inhibition recovers phosphorylation status from an atrophic state in terms of the five marker proteins. They are listed in the Additional file 2: Table S1 with the number of markers recovered. For example, we plotted the simulation results in the reference, atrophic and PTPRS-inhibited states. The PTPRS is a member of the protein tyrosine phosphatase (PTP) that is determined as one of the 37 potential therapeutic targets (Fig. 8). To avoid a crowded figure, we only depicted simulation results of two places (SMAD3 and PTPRS) instead of all 1985 places. SMAD3 is one of the five marker proteins, and we noticed that its phosphorylation status is greater in atrophic state compared to the reference state, i.e., hyper-phosphorylation, in accordance with the Western blotting experiments. Also, its status decreased in the PTPRS-inhibited state compared to the atrophic state, which indicates the recovery from the atrophic to reference state, which is a condition for a therapeutic target in this study. To obtain the status in the PTPRS-inhibited state, we averaged three simulation results. The phosphorylation status of PTPRS is zero in the inhibited state.

### Enrichment analysis to GO terms and KEGG pathways

Functional enrichment tests were performed on the 37 potential therapeutic targets against Gene Ontology (GO) terms and KEGG pathways [17, 28], and their enriched terms (adjust *p*-value < 0.001) were specified in Additional file 2: Table S2. We noticed that the enriched GO terms include several actin-related terms such as regulation of actin cytoskeleton organization, regulation of actin filament bundle assembly, and regulation of actin filament-based processes. Actin is one of the main structural proteins in muscle, and it switches on the serum-response factor (SRF) pathway that has a role in muscle development and maintenance. Therefore, mutations in the actin protein lead to alterations in the SRF pathway that could promote muscle cell degeneration and cause myopathy or muscular dystrophy [29]. The enriched KEGG pathways also include one actin-related pathway, i.e., regulation of actin cytoskeleton, and one muscle contraction pathway, i.e., vascular smooth muscle contraction.

### Evaluation with genes associated with muscle atrophy in the CTD

For an quantitative evaluation for the 37 potential targets, we generated a list of genes associated with muscle atrophy (MESH: D009133) from the Comparative Toxicogenomics Database (CTD) [16]. As the first evaluation, we obtained the three proteins, i.e. AKT1, IGF1R, and GSK3B, explicitly described as therapeutic targets of the muscle atrophy, and they were compared to the 37 potential targets via the hypergeometric test, which yields *p*-value of 0.034 (Additional file 2: Table S3). Then, we increased the size of the gold standard set by adding inferred genes in CTD up to 40 genes. To this end, the muscle atrophy associated genes in CTD are sorted by their inference scores, and we determined 20 (z-score > 1.43), 25 (z-score > 1.15), 30 (z-score > 0.89), 35 (z-score > 0.83), and 40 (z-score > 0.78) among 331 considered genes as the gold standard. The hypergeometric tests were performed on the 37 potential therapeutic targets with the five gold standards, and their results are depicted in Table 1 (refer to Additional file 2: Table S3 for more detail description). As a result, three of five comparisons produced significant results (p-value < 0.05).

**Table 1:** Results of the hypergeometric test compared to muscle atrophy-associated genes from the CTD

# of muscle atrophy associated genes from CTD	p-value of hypergeometric test
20 (z-score > 1.43)	0.172
25 (z-score > 1.15)	0.046
30 (z-score > 0.89)	0.036
35 (z-score > 0.83)	0.028
40 (z-score > 0.78)	0.059

Here, the inferred genes in CTD are genes associated with muscle atrophy, which include not only genes curing the disease (therapeutic targets) but also genes causing the disease (causing genes) as well as genes effected by the disease (effected genes). It can be a reason for not very high predictive power. In addition, an imperfect network model also can be an explanation of this low performance because there are still unknown phosphorylation reactions in a cell.

### Comparison to the shortest path analysis

Various types of proximity measures in biological networks have been developed to elucidate drug efficacies or mechanisms [30]. Among them, the shortest path analysis is typical and most widely used measure. For example, recently, the shortest path analysis has been applied on multi-level biological networks for an inference of unknown drug-disease relationship [31]. In this section, we compared the potential targets by Petri net simulation to those inferred by the shortest path analysis (SPA). To this end, we computed the average shortest path length from each of the 331 candidate proteins to the five marker proteins, and then, we selected the top 55 genes resulting in the shortest length, which are determined as atrophy-related genes by the SPA. We tried to get 37 genes, which is the same number of genes selected in this study, for a fair comparison; however, 44 genes from 12th to 55th ranked genes have the same shortest path length. A hypergeometric test between the two sets of genes (the 37 genes by Petri net and 55 genes by SPA) showed a non-significant result with *p*-value 0.136. This indicates that the Petri net-based potential targets are not connected to the marker proteins within a short length. We noticed that their shortest path lengths yield a range from 2 to 5 and their average is 3.07, which is larger than the average shortest path length of all 331 candidate proteins, 3.06. Among the 37 Petri net-based targets, 15 proteins are longer in length than the overall average, 3.06. It could be frustrating because our results are not reproduced in a shortest path analysis, which is one of the most popular static network analysis. However, on the other hand, we think that Petri net simulation can characterizes atrophy-related gene sets, which cannot be predicted by the shortest measure.

### Literature evidence

We validated several predicted therapeutic target genes by literature searches. BMPR1B is a member of the bone morphogenetic protein (BMP) receptor family, which is reported as a positive regulator of muscle mass in Winbanks’ work [32]. Rho-associated kinase (ROCK) is a regulator of the actomyosin cytoskeleton that induces contractile force generation. Hudson’s group identified that ROCK is highly correlated with the phosphorylation level of myosin phosphatase that regulates myosin light chains [33]. Moreover, in Li’s experiments, an inhibitor of ROCK2 attenuated the Angiotensin II induced contraction in human airway smooth muscle cells [34]. LEPR is a receptor of leptin hormone that regulates adipose-tissue mass, and its expression is highly up-regulated in the condition of skeletal muscle disuse atrophy in humans [35].

## Discussion

Among several well-known simulation tools including Petri net, Random walk and ordinary differential equation (ODE), we consider that Petri net is the most appropriate tool for this work. The size of the constructed phosphorylation network is so large (7283 interactions) that we cannot employ ODE, whose kinetic parameters are incomplete. Additionally, it is not possible to employ Random walk for this study that particularly focuses on phosphorylation status, which involves two types of interactions and two types of nodes.

We expected that myostatin direct targets, such as ACVR1 and ACVR1B, would be included in the predicted therapeutic targets because their inhibition produces almost the same effect as the inhibition of myostatin, which should recover the phosphorylation status from an atrophic state for the all five marker proteins; however, ACVR1 and ACVR1B are excluded. When they were inhibited, the numbers of markers recovered are 5, 5, 3 for ACVR1 and 4, 5, 3 for ACVR1B for the three simulations in the gene-inhibited state, which does not satisfy the selection criteria for potential therapeutic targets. We recognize that the criteria should be modified so as not to miss such obvious targets, and this will be carried out in further work.

## Conclusions

In this study, we suggested a new approach to predict potential therapeutic targets for muscle atrophy based on the phosphorylation status of the reference, atrophic and gene-inhibited states, which were simulated by Petri net. The simulation results between the reference and atrophic states were validated with Western blotting experiments detecting five marker proteins. We generated a list of the potential therapeutic targets whose inhibition recovers the five abnormally phosphorylated proteins in the atrophic state, and they were evaluated by various approaches, such as GO terms, the literature, and genes associated with muscle atrophy. We expect the new proposed strategy can provide an understanding of phosphorylation status in muscle atrophy and provide assistance towards identifying its therapeutics.

## Additional files

Additional file 1: **Figure S1.** An average of rank correlations among ten simulations in pair-wise manner for each of eleven thresholds. We selected the enabling threshold 35 that show the highest rank correlation in the reference and atrophic states. (DOCX 51 kb)

Additional file 2: **Table S1.** The 37 characterized therapeutic targets. **Table S2.** The enriched GO terms and KEGG pathways. **Table S3.** Hypergeometric test with gold standard from CTD. (XLSX 19 kb)

## References

[1] Cohen S, Nathan JA, Goldberg AL (2015). Muscle wasting in disease: molecular mechanisms and promising therapies. Nat Rev Drug Discov.

[2] Coats AJ (2002). Origin of symptoms in patients with cachexia with special reference to weakness and shortness of breath. Int J Cardiol.

[3] Workeneh BT, Mitch WE (2010). Review of muscle wasting associated with chronic kidney disease. Am J Clin Nutr.

[4] Evans WJ, Morley JE, Argilés J, Bales C, Baracos V, Guttridge D, Jatoi A, Kalantar-Zadeh K, Lochs H, Mantovani G (2008). Cachexia: a new definition. Clin Nutr.

[5] Lok C (2015). Cachexia: the last illness. Nature.

[6] Molfino A, Amabile MI, Rossi Fanelli F, Muscaritoli M (2016). Novel therapeutic options for cachexia and sarcopenia. Expert Opin Biol Ther.

[7] Balazki P, Lindauer K, Einloft J, Ackermann J, Koch I (2015). MONALISA for stochastic simulations of petri net models of biochemical systems. BMC Bioinformatics.

[8] Lee D-Y, Zimmer R, Lee SY, Park S (2006). Colored petri net modeling and simulation of signal transduction pathways. Metab Eng.

[9] Ruths D, Muller M, Tseng J-T, Nakhleh L, Ram PT (2008). The signaling petri net-based simulator: a non-parametric strategy for characterizing the dynamics of cell-specific signaling networks. PLoS Comput Biol.

[10] Jin G, Zhao H, Zhou X, Wong ST (2011). An enhanced petri-net model to predict synergistic effects of pairwise drug combinations from gene microarray data. Bioinformatics.

[11] Karin M, Hunter T (1995). Transcriptional control by protein phosphorylation: signal transmission from the cell surface to the nucleus. Curr Biol.

[12] Cohen P (2001). The role of protein phosphorylation in human health and disease. Eur J Biochem.

[13] Stitt TN, Drujan D, Clarke BA, Panaro F, Timofeyva Y, Kline WO, Gonzalez M, Yancopoulos GD, Glass DJ (2004). The IGF-1/PI3K/Akt pathway prevents expression of muscle atrophy-induced ubiquitin ligases by inhibiting FOXO transcription factors. Mol Cell.

[14] Hasselgren PO (2007). Ubiquitination, phosphorylation, and acetylation—triple threat in muscle wasting. J Cell Physiol.

[15] Lokireddy S, Wijesoma IW, Bonala S, Wei M, Sze SK, McFarlane C, Kambadur R, Sharma M (2012). Myostatin is a novel tumoral factor that induces cancer cachexia. Biochem J.

[16] Davis AP, Grondin CJ, Johnson RJ, Sciaky D, King BL, McMorran R, Wiegers J, Wiegers TC, Mattingly CJ (2017). The comparative toxicogenomics database: update 2017. Nucleic Acids Res.

[17] Kanehisa M, Goto S (2000). KEGG: kyoto encyclopedia of genes and genomes. Nucleic Acids Res.

[18] Duan G, Li X, Köhn M (2015). The human DEPhOsphorylation database DEPOD: a 2015 update. Nucleic Acids Res.

[19] Hu J, Rho H-S, Newman RH, Zhang J, Zhu H, Qian J (2014). PhosphoNetworks: a database for human phosphorylation networks. Bioinformatics.

[20] Uhlén M, Fagerberg L, Hallström BM, Lindskog C, Oksvold P, Mardinoglu A, Sivertsson Å, Kampf C, Sjöstedt E, Asplund A (2015). Tissue-based map of the human proteome. Science.

[21] Schlessinger J (2000). Cell signaling by receptor tyrosine kinases. Cell.

[22] Cohen P (2000). The regulation of protein function by multisite phosphorylation–a 25 year update. Trends Biochem Sci.

[23] Heinrich R, Neel BG, Rapoport TA (2002). Mathematical models of protein kinase signal transduction. Mol Cell.

[24] Argilés JM, Busquets S, Stemmler B, López-Soriano FJ (2014). Cancer cachexia: understanding the molecular basis. Nat Rev Cancer.

[25] Lokireddy S, Mouly V, Butler-Browne G, Gluckman PD, Sharma M, Kambadur R, McFarlane C (2011). Myostatin promotes the wasting of human myoblast cultures through promoting ubiquitin-proteasome pathway-mediated loss of sarcomeric proteins. Am J Physiol Cell Physiol.

[26] Trendelenburg AU, Meyer A, Rohner D, Boyle J, Hatakeyama S, Glass DJ: Myostatin reduces Akt/TORC1/p70S6K signaling, inhibiting myoblast differentiation and myotube size. American Journal of Physiology-Cell Physiology 2009;296(6):C1258–C1270. 10.1152/ajpcell.00105.2009 19357233

[27] Sriram S, Subramanian S, Sathiakumar D, Venkatesh R, Salerno MS, McFarlane CD, Kambadur R, Sharma M (2011). Modulation of reactive oxygen species in skeletal muscle by myostatin is mediated through NF‐κB. Aging cell.

[28] Ashburner M, Ball CA, Blake JA, Botstein D, Butler H, Cherry JM, Davis AP, Dolinski K, Dwight SS, Eppig JT (2000). Gene ontology: tool for the unification of biology. Nat Genet.

[29] Visegrady B, Machesky LM (2010). Myopathy-causing actin mutations promote defects in serum-response factor signalling. Biochem J.

[30] Guney E, Menche J, Vidal M, Barábasi A-L (2016). Network-based in silico drug efficacy screening. Nat Commun.

[31] Yu H, Jung J, Yoon S, Kwon M, Bae S, Yim S, Lee J, Kim S, Kang Y, Lee D (2017). CODA: integrating multi-level context-oriented directed associations for analysis of drug effects. Sci Rep.

[32] Winbanks CE, Chen JL, Qian H, Liu Y, Bernardo BC, Beyer C, Watt KI, Thomson RE, Connor T, Turner BJ (2013). The bone morphogenetic protein axis is a positive regulator of skeletal muscle mass. J Cell Biol.

[33] Hudson CA, Heesom KJ, Bernal AL (2012). Phasic contractions of isolated human myometrium are associated with rho-kinase (ROCK)-dependent phosphorylation of myosin phosphatase-targeting subunit (MYPT1). Mol Hum Reprod.

[34] Li N, Cai R, Niu Y, Shen B, Xu J, Cheng Y (2012). Inhibition of angiotensin II-induced contraction of human airway smooth muscle cells by angiotensin-(1-7) via downregulation of the RhoA/ROCK2 signaling pathway. Int J Mol Med.

[35] Chen Y-W, Gregory CM, Scarborough MT, Shi R, Walter GA, Vandenborne K (2007). Transcriptional pathways associated with skeletal muscle disuse atrophy in humans. Physiol Genomics.

