# Supplementary material for: Petri net-based prediction of therapeutic targets that recover abnormally phosphorylated proteins in muscle atrophy
Source: BMC Syst Biol. 2018 Mar 5;12:26. doi: 10.1186/s12918-018-0555-0 (PMC5838966; doi:10.1186/s12918-018-0555-0)
Supplement: Supplementary file 1 — Figure S1. An average of rank correlations among ten simulations in pair-wise manner for each of eleven thresholds. We selected the enabling threshold 35 that show the highest rank correlation in the reference and atrophic states. (DOCX 51 kb) [file 12918_2018_555_MOESM1_ESM.docx]

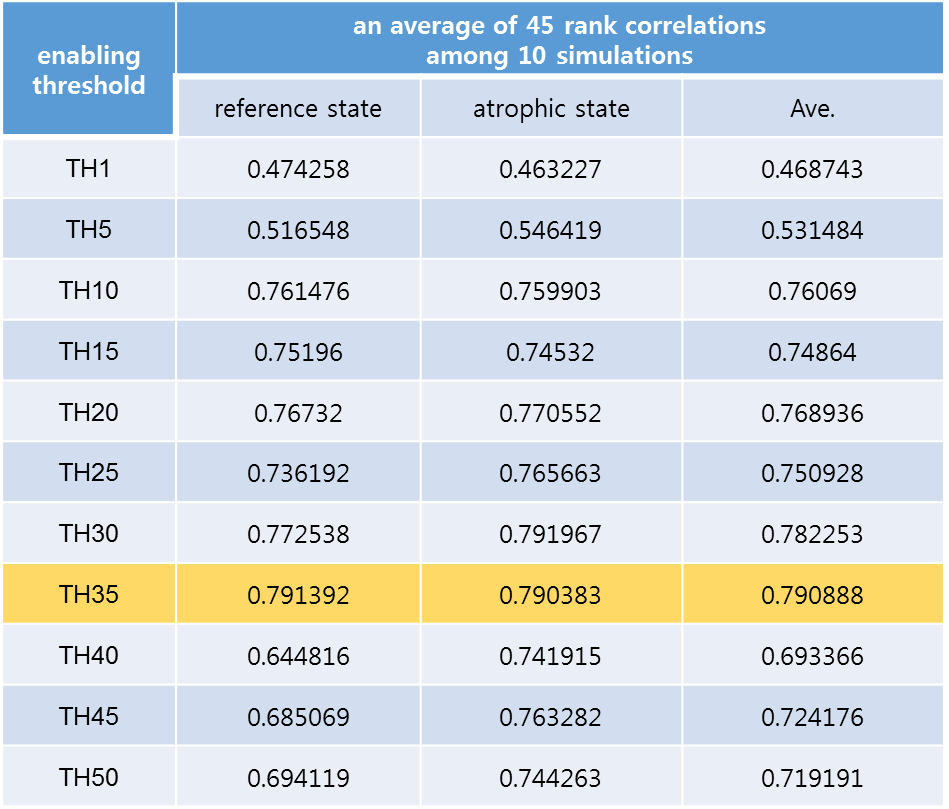


Fig. S1. An average of rank correlations among ten simulations in pair-wise manner for each of eleven thresholds. We selected the enabling threshold 35 that show the highest rank correlation in the reference and atrophic states.
